# An ankylosaur larynx provides insights for bird-like vocalization in non-avian dinosaurs

**DOI:** 10.1038/s42003-023-04513-x

**Published:** 2023-02-15

**Authors:** Junki Yoshida, Yoshitsugu Kobayashi, Mark A. Norell

**Affiliations:** 1 Hokkaido University Museum, Sapporo, Hokkaido, Japan; 2 Fukushima Museum, Aizu-wakamatsu, Fukushima, Japan; 3 American Museum of Natural History, New York, NY USA

**Keywords:** Palaeontology, Palaeoecology

## Abstract

A voice box (larynx) is unique for tetrapods and plays functional roles in respiration, airway protection, and vocalization. However, in birds and other reptiles, the larynx fossil is extremely rare, and the evolution of this structure remains largely unknown. Here we report the fossil larynx found in non-avian dinosaurs from ankylosaur *Pinacosaurus grangeri*. The larynx of *Pinacosaurus* is composed of the cricoid and arytenoid like non-avian reptiles, but specialized with the firm and kinetic cricoid-arytenoid joint, prominent arytenoid process, long arytenoid, and enlarged cricoid, as a possible vocal modifier like birds rather than vocal source like non-avian reptiles. Although bird-unique vocal source (syrinx) have never been reported in non-avian dinosaurs, *Pinacosaurus* could have employed bird-like vocalization with the bird-like large, kinetic larynx. This oldest laryngeal fossil from the Cretaceous dinosaur provides the first step for understanding the vocal evolution in non-avian dinosaurs toward birds.

## Abstract (web summary)

The earliest larynx discovered in fossil dinosaurs indicates that non-avian dinosaurs may have had bird-like vocalization.

## Introduction

A hyolaryngeal apparatus (tongue and voice box) is a key evolutionary trait in tetrapods and is associated with their feeding, respiration, and vocalization^1–12^. Particularly, the larynx is an entrance to the tracheal passage and is involved in vocalization (e.g., sound communication). Among modern archosaurs, the hyolaryngeal apparatuses of crocodilians and birds differ both anatomically and functionally^7,10^. In crocodilians, the larynx produces sound as a vocal source^7^. In birds, the syrinx produces sound at the posterior end of the trachea, and increases vocal efficiency as a vocal source^10,13–15^, while the larynx functions as a part of the vocal tract^16^. Although hyoids are sometimes preserved as fossils and the evolution of the hyoid has been previously discussed^17,18^, no larynx has been reported in extinct non-avian reptiles and the evolution of this organ is largely unknown^14^. This is probably because the larynx is cartilaginous in all tetrapods except neognath birds. Preserved hyoids in non-avian dinosaurs are only ceratobranchials except in the theropod *Carnotaurus*, *Microraptor*, and *Confuciusornis* (basihyal and ceratobranchial 1)^18,19^ and some ankylosaurs (basihyal, and ceratohyal, and ceratobranchial 1 in *Saichania chulsanensis* and paraglossal, ceratobranchial 1, ceratobranchial 2, and epibranchial in *Pinacosaurus grangeri*)^17,20^. *Pinacosaurus grangeri* (IGM100/3186) has the best-preserved hyolaryngeal apparatus in non-avian dinosaurs. Here we examine the hyolaryngeal apparatus of *Pinacosaurus* (IGM100/3186) and provide the first description of the larynx of non-avian dinosaurs with its comparisons to modern reptiles and birds.

## Results

### Description

**Fig. 1:**
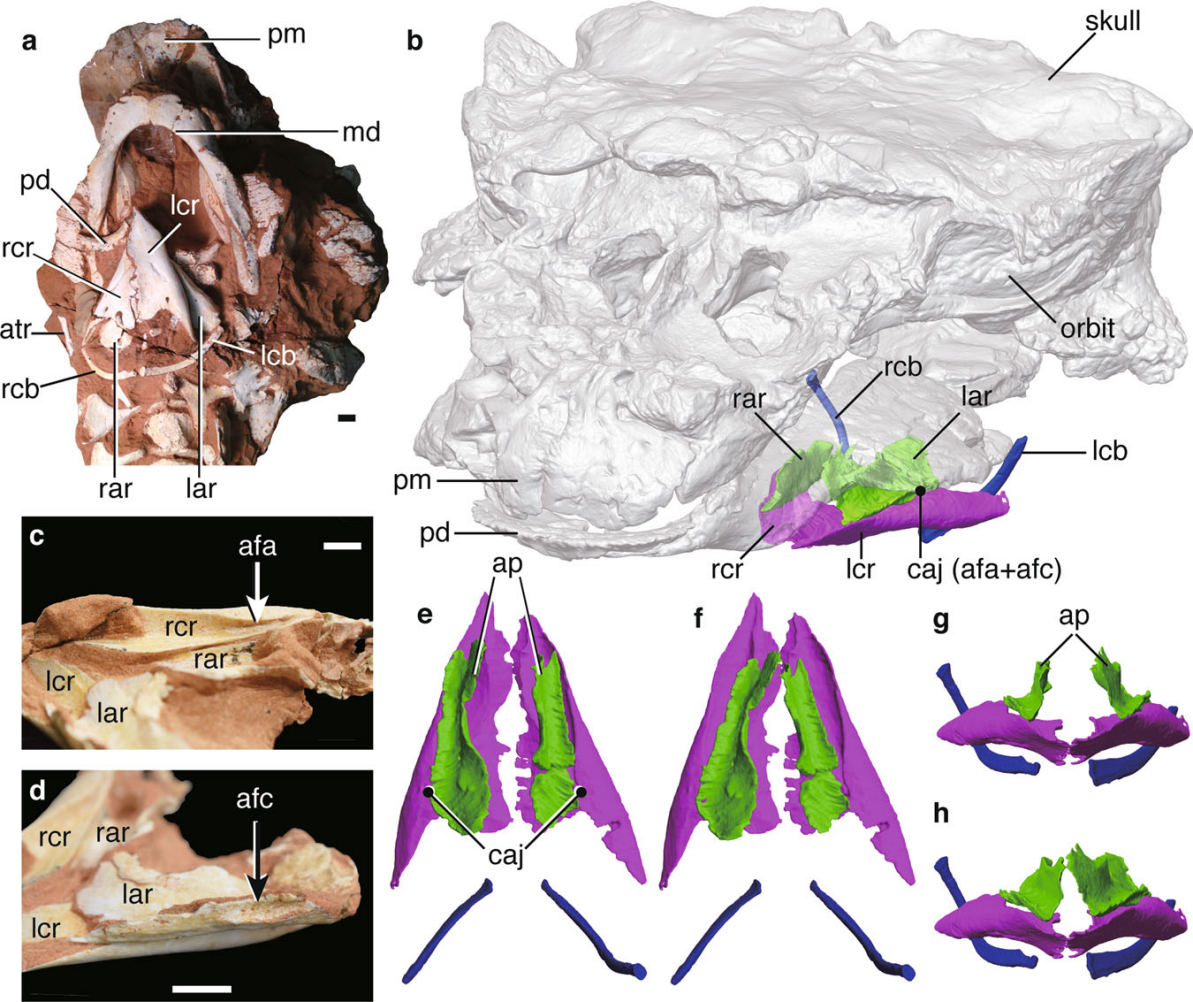
in situ hyolaryngeal apparatus and skull of *Pinacosaurus*. **a** Ventral view and **b** 3D reconstruction of skull, mandible, and hyolaryngeal apparatus in left oblique view. **c** Crico-aryteniod joint of right cricoid in medial view. **d** The joint of left arytenoid in dorsolateral view. **e** Arytenoid position in glottal opening and **f** glottal closing in anterior views. **g** Arytenoid position in glottal opening and **h** glottal closing in dorsal views. Abbreviations: afa, articular facet for arytenoid; afc, articular facet for cricoid; ap, arytenoid process; atr, atlas rib; caj, crico-arytenoid joint; lcb, left ceratobranchial; lcr, left cricoid; md, mandible; pm, premaxilla; pd, predentary; rar, right arytenoid; rcb, right ceratobranchial; rcr, right cricoid. Scale bars, 1 cm. Photograph by Michael D’Emic and edited by JY in **a**.

*Pinacosaurus* (IGM100/3186) preserves two laryngeal (cricoids and arytenoids) and one hyoid (ceratobranchials) elements (Fig. 1, Supplementary Note 1, Supplementary Figs. 1, 2, and 6). The symmetrical arrangement of these elements along the midline and the dorsal position of the arytenoid to the cricoid indicate that the hyolaryngeal apparatus is preserved in almost life position (Fig. 1a). Both cricoids (Fig. 1e, h) are complete but remain unfused at the midline, while those in the ankylosaurid *Saichania* are fused into a single element^20^. This probably has to do with the somatic immaturity of IGM100/3186^17^. The cricoids are the largest bones of the hyolaryngeal apparatus. They are at least 64.7 mm wide and 65% of the mandible width. The cricoid is pointed anteriorly and widely expands posteriorly, with a concave posterior margin, forming an arrow-head shape in dorsal view. The anterior tip of the cricoid projects beyond the arytenoids, which may be an attachment site for the glottal constrictor muscle like ostrich^10^. The lateral edge of the cricoid body curls dorsally to form the cricoid wing. A dorsomedially projecting thin lamina extends anteroposteriorly from the anterior apex of the cricoid body and ends medial to the cricoid wing. At the intersection of the cricoid lamina and wing, a shallow groove (~2 cm long) forms an articular facet for the arytenoid (Fig. 1c). The dorsal surface above the groove has a rugose surface, indicating a cartilaginous pad for a joint with the arytenoid. A minute foramen lies near the posterior edge of the cricoid body on each side (Fig. 1a and Supplementary Fig. 1d), similar to two posterior notches in the cricoid of *Alligator mississippiensis*^7^ and the angular foramen on the cricoid in several birds (Supplementary Fig. 2) and chicken embryos^21^.

A pair of arytenoids lies dorsal to the cricoid (Fig. 1a, b, e–h). Those are anteroposteriorly long and bear short dorsolateral and long dorsomedial wings, which are J-shaped in cross-section (Fig. 1). The dorsomedial wing is thin, and its anterior third projects dorsally to form the arytenoid process as in turtles^22^ and birds^10,23^. The arytenoid process is an attachment site for m. dilator laryngis, which opens the glottis in crocodilians^7,9^ and birds^10,11^. The arytenoid process of *Pinacosaurus* is not as prominent as that of *Saichania*^20^ (Supplementary Fig. 3). This is probably due to its relative somatic immaturity. Like birds the arytenoid process ossifies late in ontogeny^10^. The dorsolateral wing is less developed than the dorsomedial wing. Furthermore, the lateral surface of the dorsolateral wing bears a ridge with a rugose surface that articulates with a corresponding rugose surface lying at the constriction of the above-mentioned cricoid groove (Fig. 1d). This forms the ridge-and-groove articulation between the cricoid and arytenoid. This unique morphology of the articular facet is not found in other reptiles including birds.

The paired ceratobranchials have proximal ends that lie medially along the midpoint axis of the body, while their distal ends splay laterally (Fig. 1a). The ceratobranchials were placed close to each other, and the distance between the proximal ends is about 2 mm. This is much smaller than the width of the cricoid. The ceratobranchial is slender, slightly curved ventrally, 36 mm long, about the half-length of the mandible, and ~70 % of the cricoid length (Fig. 1b, e–h).

### Morphometrics

**Fig. 2:**
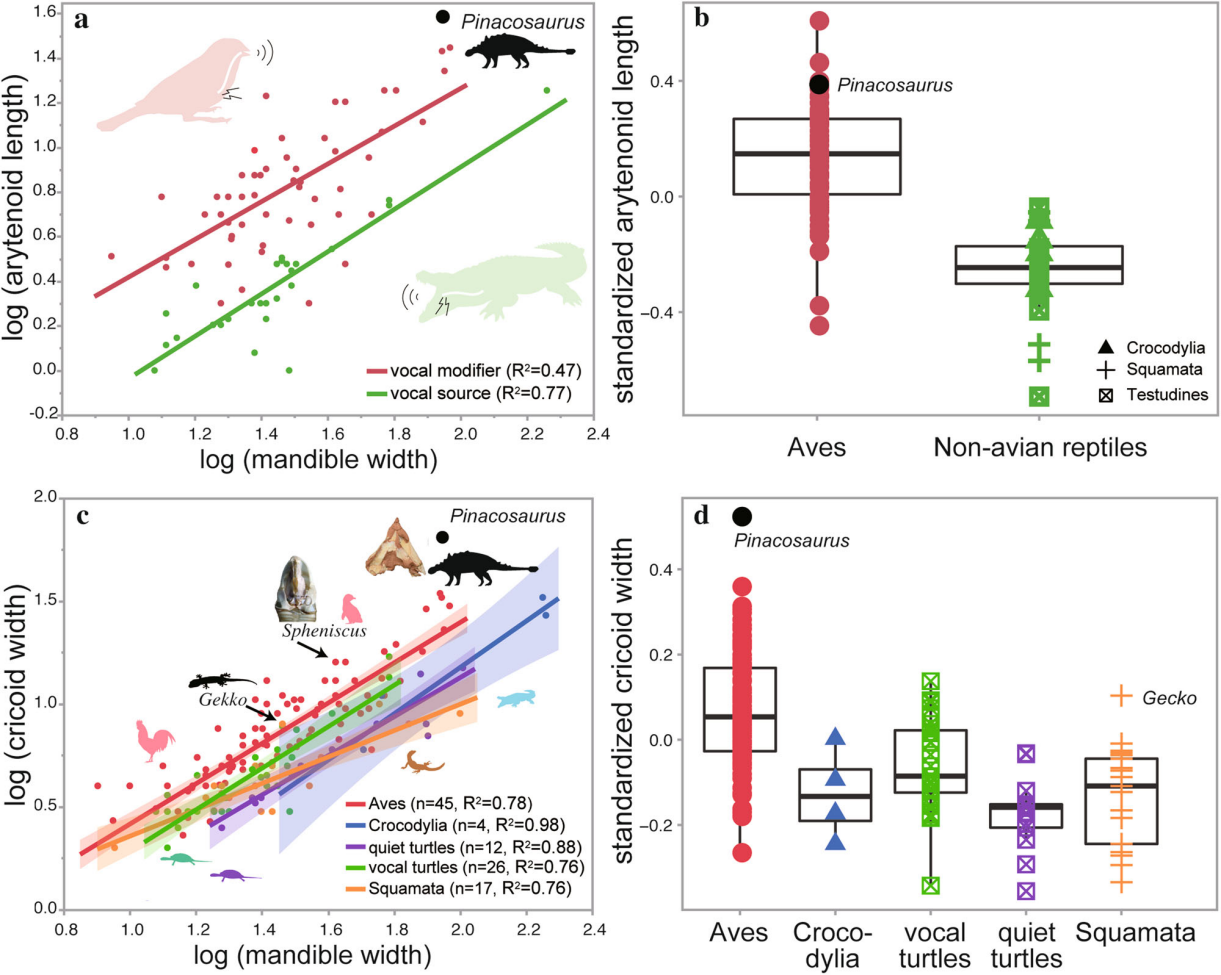
Morphometrics of larynges of reptiles and birds. **a** Size of arytenoid (length) to mandible width in reptiles (*n* = 31) and birds (*n* = 58). (**b**) Box-plot of the standardized arytenoid length between birds and non-avian reptiles. **c** Size of cricoid (width) to mandible width in reptiles (*n* = 62) and birds (*n* = 90). **d** Box-plot of the standardized cricoid width among birds and non-avian reptiles. Linear regression lines showing the 95% confidence intervals of Ordinary Least Squares in **a**, **c**. Silhouettes from Phylopic (http://phylopic.org) by Andrew Farke, Dysalatornis, Michael Keesey, and Steven Traver.

Arytenoids are short in larynges of non-dinosaurian reptiles and long in dinosaurs, such as *Pinacosaurus* and birds (Fig. 2a). A linear regression analysis shows that the arytenoid length is positively correlated with mandible width (Fig. 2a). However, a distinction in arytenoid size between a group of vocal source (i.e., reptiles) and that of non-vocal source but vocal modifier (i.e., birds) is also supported by student’s t-test of residuals of arytenoid length standardized by mandible width (*t* = −9.556, df = 87, *p* < 0.0001, Fig. 2b).

Cricoid width is large in a gecko, vocal turtles, birds, and *Pinacosaurus*, and the cricoid width is positively correlated with mandible width (Fig. 2c). Birds differ in relative cricoid width from non-avian reptiles, which is statistically supported by Tukey HSD test of residuals of cricoid width standardized by mandible width (birds vs. crocodilians, *p* = 0.025; birds vs. squamates, vocal turtles, and quiet turtles, *p* < 0.0001, Fig. 2d).

## Discussion

### Fossil record and ossification of larynx in Archosauria

**Fig. 3:**
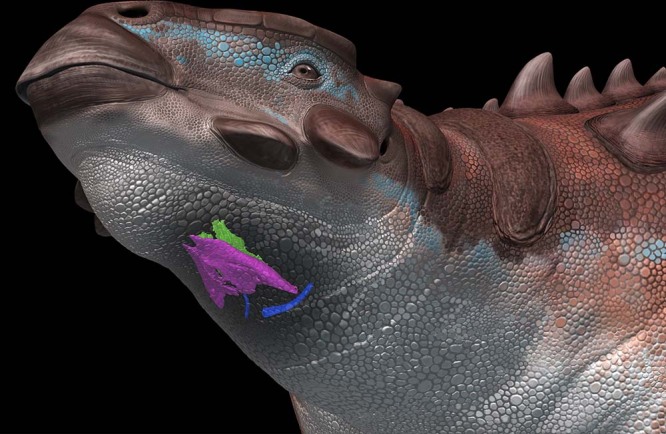
Hyolaryngeal apparatus of *Pinacosaurus* in a life restoration. Cricoid (purple), arytenoid (green), and ceratobranchial (blue) are depicted. Artwork by Tatsuya Shinmura.

A fossilized larynx has not been reported in non-avian archosaurs and has been even rarely preserved in fossil birds. The oldest larynx in the fossil record was the cricoid of the anseriform *Presbyornis* (56–46 million years ago) from the Lower Eocene Green River Formation in Wyoming, USA^24^. Some hyolaryngeal elements of *Pinacosaurus* from the Cretaceous Djadokhta Formation and *Saichania* from the Cretaceous Baruungoyot Formation in Mongolia (84–72 million years ago), which were originally identified as the hyoids, paraglossal and ceratobranchial 1^17,20^, are actually the larynx (cricoid and arytenoid) (Supplementary Fig. 3). Hence, this is the first report of the larynx in non-avian tetrapods during pre-Cenozoic eras (Fig. 3).

The ankylosaurs, *Pinacosaurus* (IGM100/3186) and *Saichania* (IGM100/151), share the ossification of cricoid and arytenoid (Supplementary Fig. 3) as in birds (Supplementary Fig. 2 and (ref. ^25^)). This indicates that the larynx is ossified in some non-avian dinosaurs. It still remains possible that the laryngeal ossification is ankylosaurid synapomorphy, or absent in other fossils simply due to poor preservation during fossilization or collection and preparation artifacts. In terms of development, the ossified larynx in the juvenile *Pinacosaurus* (IGM1000/13186) is similar to the early developmental condition in birds^26^.

### Laryngeal morphology and vocalization of *Pinacosaurus*

The arytenoid process and firm cricoid-arytenoid joint in *Pinacosaurus* enable horizontal rotation of the arytenoid to open and close the glottis (Fig. 1e, h). This architecture could have controlled the laryngeal configuration and air pressure across the glottis. Only in birds does the arytenoid exhibit such a distinct arytenoid joint, in contrast to a connective tissue bridge as in other reptiles (Supplementary Figs. 4 and5). Furthermore, longer arytenoids in *Pinacosaurus* and birds than in non-dinosaurian reptiles (Fig. 2b) could result in a larger attachment area and a longer lever arm of the dilator muscle than the other reptiles, which enable them to open the glottis easily rather than close. In birds, the laryngeal glottis opens when it emits sounds produced by vibratory tissues in the syrinx^27^, while, in non-dinosaurian reptiles such as crocodilians and turtles^9,28^, the glottis is almost closed because it efficiently produces sound by the vibration of the vocal folds around the glottis dorsal or anterodorsal to arytenoids^29^. Therefore, the combination of the large arytenoid process, the firm cricoid-arytenoid joint, and the long arytenoids have likely allowed *Pinacosaurus* to open the glottis easily as in birds and may be used for avian-like airflow regulation, especially modifying a sound.

The larynges of dinosaurs (*Pinacosaurus* and birds) also show larger cricoids than non-dinosaurian reptiles (Fig. 2c). Similarly, vocal reptiles, such as gekkotans^30,31^ and some turtles (testudinids, trionychids, emydids, and batagurids)^28,32,33^, possess larger cricoids than non-vocal reptiles (Fig. 2c). The cricoid enlargement is common among the vocal groups, which can be explained by the flared end of the trachea as a part of the vocal tract and likely increases radiative efficiency^34^. Therefore, the larynx of *Pinacosaurus* may have been actively vocalized and associated with loud and explosive calls as in vocal reptiles and birds^30,31,35,36^.

In tetrapods, three different functions of larynx have been suggested: (1) airway protection (a barrier to foreign materials entering the trachea), (2) respiratory modulation (opening the glottis during respiration), and (3) acoustic communication (generation and modulation of sound)^37,38^. The large and kinetic larynx of *Pinacosaurus*, characterized by the large cricoid, firm cricoid-arytenoid joint, prominent arytenoid process, and long arytenoid, could be related to airflow regulation which functions in respiratory modulation and acoustic communication. Particularly, the four features of *Pinacosaurus* resemble that of birds, indicating that the kinetic larynx of *Pinacosaurus* is similar to birds such as parrots and passerines, which can largely change the configuration of the laryngeal cavity probably related to the complexity of vocalization^11^. Meanwhile, airway protection cannot explain these morphologies since it does not require a large and kinetic larynx for glottal closure. Therefore, the larynx of *Pinacosaurus* was specialized for opening the glottis and possibly a sound modifier with other vocal tracts such as the trachea and oral, esophageal, and pharyngeal cavities. Its vocalization might be related to courtship, parental call, predator defense, and territorial calls, as in modern archosaurs, crocodilians and birds^35,36^.

In birds, the power source for their vocalization is the anterior air sac system, which shrinks during exhalation^39^. Although osteological features for anterior air sac are absent in ankylosaurs and other ornithischian dinosaurs, their rib joint arrangement of the thoracic cavities are bird-like architecture^40^, indicating the presence of a dorsally immobilized lung like birds^40^ and anterior air sacs like saurischian dinosaurs (*Saltasaurus*^41^, *Aerosteon*, and also birds^42^) as a power source for sound production in non-avian dinosaurs. Although fossil evidence of the vocal source in non-avian dinosaurs has never been found so far, since *Pinacosaurus* is similar to birds in having a large, kinetic larynx and immobile lungs, this dinosaur likely possessed a non-laryngeal vocal source and enhanced their vocal activity and sound communication like modern birds.

### Hyolaryngeal evolution of Archosauria

**Fig. 4:**
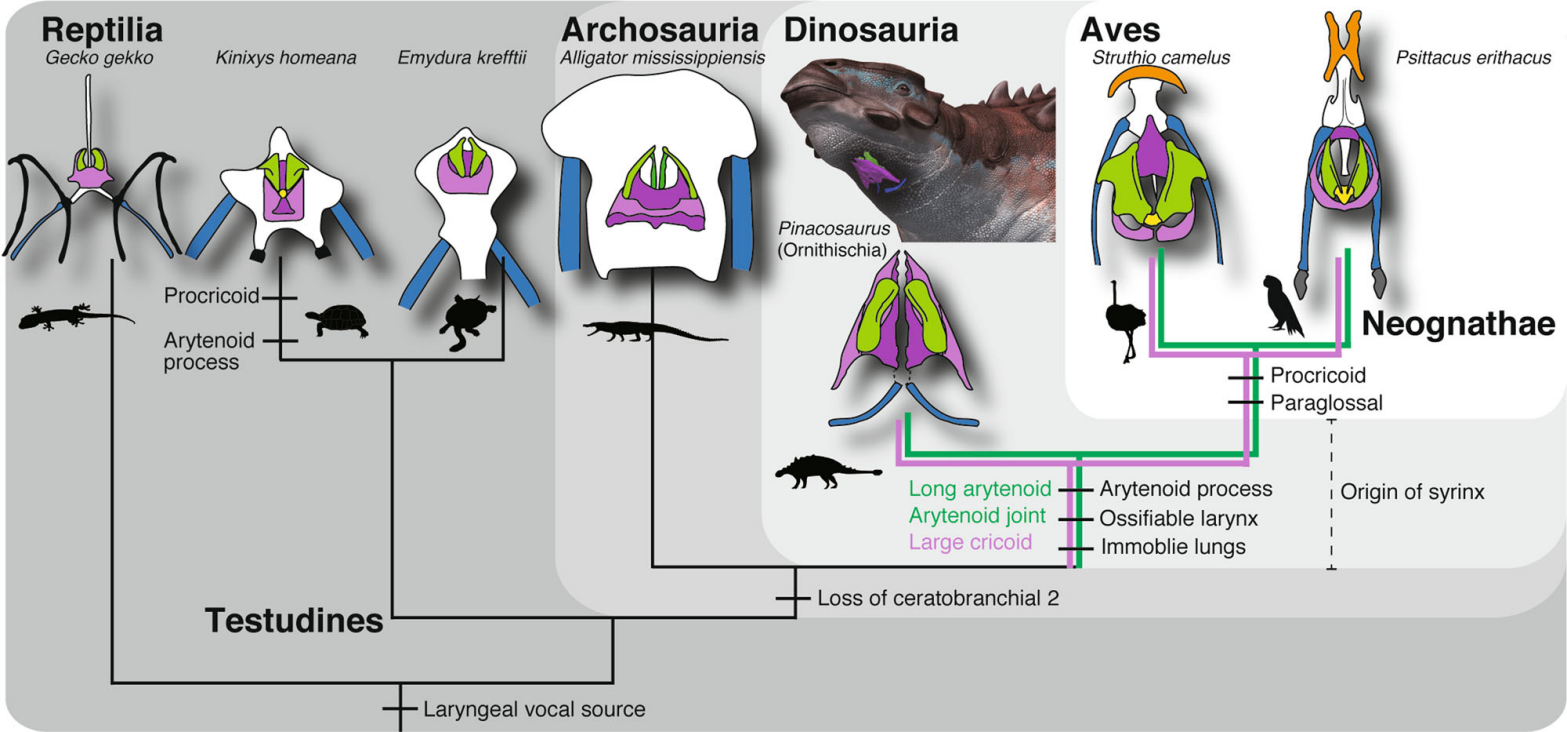
Evolution of hyolaryngeal apparatus in Archosauria. Ceratobranchial (blue), basihyal (white), paraglossal (orange), cricoid (purple), arytenoid (green), and procricoid (yellow). Colored lines indicate presence of the characters. A phylogenetic relationship of Testudines follows (ref. ^43^). A flesh-out reconstruction of *Pinacosaurus* with its hyolaryngeal apparatus (illustration by Tatsuya Shinmura). Silhouettes from Phylopic (http://phylopic.org) by Andrew Farke, Aline Ghilardi, Scott Hartman, Lukasiniho, Steven Traver, and Yan Wong.

IGM100/3186 newly illustrates the evolution of diverse hyolaryngeal apparatus in Archosauria (Fig. 4). Ceratobranchial 2 is commonly present in non-archosaurian reptiles, and lost in Archosauria^18^. The hyolaryngeal apparatus of crocodilians is composed of four elements (arytenoid, cricoid, basihyal, and ceratobranchial 1), where all are cartilaginous except ceratobranchial 1. *Pinacosaurus*, an ornithischian dinosaur, retained the same hyolaryngeal elements as crocodilians. Yet, *Pinacosaurus* shows many shared characters with birds in the arrangement and morphology of the larynx (Fig. 4), such as, ossification of the larynx, an arytenoid process, a firm articular joint between the cricoid and arytenoid, long arytenoids, large cricoids, and a pointed tip of the cricoid (Fig. 1 and Supplementary Fig. 1). Paraglossal, procricoid, and epibranchial of birds are presumably retained in saurischian lineage (Fig. 4). As mentioned above, morphologies of the larynges indicate that *Pinacosaurus* did not use the larynx as a sound source like non-avian reptiles, but probably worked as a sound modifier like birds. We propose that bird-like vocalization have likely appeared in non-avian dinosaurs before the advent of Aves.

## Methods

### Description of ankylosaur specimen

Ankylosaur dinosaur *Pinacosaurus* (IGM100/3186) is described as hyolaryngeal apparatus of fossil archosaurs. This specimen was discovered from Ukhaa Tolgod, Mongolia and its geological age is middle Campanian of the Late Cretaceous (*17*). The specimen is better prepared for observation and measurement after the previous publication in 2015. Computed Tomography and Laser scanning are conducted in AMNH to study its morphology. We used CT images to make a reconstruction of the larynx from open data at Morphobank (http://www.morphobank.org, ID: P2101).

### Comparative study of larynx in modern and fossil skeletal specimens

For comparison, extant species of reptiles and birds (45 specimens of turtles, 14 of lizards, 4 of crocodilians, and 90 of birds) are examined qualitatively and quantitatively in American Museum of Natural History, New York, and National Museum of Nature and Science, Tokyo (Supplementary Information). We also studied the palaeognath bird skeletons, such as extant *Nothura* (AMNH10804), *Nothoprocta* (AMNH 6498, 6502), and extinct *Dinornis* (AMNH7301). Maximum transverse widths of cricoid and mandible, and maximum anteroposterior lengths of arytenoid are measured (Supplementary Fig. 1). For size normalization, all the measurements were normalized using the regression equation (Supplementary Data). For relative size comparisons, residuals of arytenoid length and cricoid width are obtained from mandible width, which are all log-transformed variables (Supplementary Data). All statistical analyses were performed using JMP Pro v.14. Linear regression analyses were conducted in two bi-plots: arytenoid length and mandible width, and cricoid and mandible widths. Student’s *t* test and Tukey HSD test were performed in the standardized arytenoid length and cricoid width.

### Reporting summary

Further information on research design is available in the Nature Portfolio Reporting Summary linked to this article.

## Supplementary information

Supplementary Material

Description of Additional Supplementary Files

Supplementary Data 1

Reporting Summary

## Data Availability

The specimen of *Pinacosaurus* (IGM100/3186) is stored in the Institute of Paleontology in Ulaanbaatar, Mongolia. CT images for a reconstruction of the hyolarynx are openly available at Morphobank (http://www.morphobank.org, ID: P2101). Measurement data are available online at the Supplementary Information.
