# Supplementary material for: An ankylosaur larynx provides insights for bird-like vocalization in non-avian dinosaurs
Source: Commun Biol. 2023 Feb 15;6:152. doi: 10.1038/s42003-023-04513-x (PMC9932143; doi:10.1038/s42003-023-04513-x)
Supplement: Supplementary file 1 — Supplementary Material [file 42003_2023_4513_MOESM1_ESM.pdf]

SUPPLEMENTARY NOTE 1

An ankylosaur larynx provides insights for bird-like vocalization in non-avian dinosaurs

Junki Yoshida[1,2*], Yoshitsugu Kobayashi[1], Mark A. Norell[3]

[1]Hokkaido University Museum, Sapporo, Hokkaido, Japan
[2]Fukushima Museum, Aizu-wakamatsu, Fukushima, Japan
[3]American Museum of Natural History, New York, NY, USA
*Corresponding author: junkiyoshida9@gmail.com

CONTENTS

- New and old identifications in hyolaryngeal apparatus of *Pinacosaurus* (IGM1000/3186)
- Full description of hyolaryngeal apparatus of *Pinacosaurus* (IGM1000/3186)
- Supplementary Figures 1 to 6
- References
- Morphometrics of larynx: arytenoid length, cricoid width, and the standardized values of those two measurements by mandible width (separate file).

Institutional abbreviations: AMNH, American Museum of Natural History, New York, USA; IGM, Institute of Geology, Ulaan Baatar, Mongolia; NSMT, National Museum of Nature and Science, Tokyo, Japan; ROM, Royal Ontario Museum, Toronto, Canada; ZSM, Zoologische Staatssammlung München, Germany

1. New and old identifications in hyolaryngeal apparatus of *Pinacosaurus* (IGM1000/3186)

We revise the identification of the elements described previously[17]: the paraglossal is re-identified as the cricoid, ceratobranchial 1 as the arytenoid, ceratobranchial 2 as ceratobranchial 1, and epibranchial as an atlas rib (Fig. 1 and Supplementary Figs. 1, 3, and 6). IGM100/3186 preserves three in situ paired symmetrical hyolaryngeal elements (cricoid, arytenoid, and ceratobranchial 1) (Fig. 1a, b). The cricoid and arytenoid are articulated and lie anterior to the ceratobranchial 1. The basihyal is not present, probably because it was unossified. Paired atlas ribs are preserved; one is located lateral to the jaw joint and another is posterior to the cricoid. Since the previous studies have never mentioned possibility of larynx in ankylosaurs[17,20], it remains equivocal whether they considered it or not. We consider possibilities of hyoids as in their studies and raise an argument to their identifications. The identification of the bones made by Hill et al.[17] worth re-considering in the following seven characters: presence of the paraglossal, absence of paraglossal-basihyal joint, medial groove in lateral margin of the paraglossal, un-ossification of the basihyal, plate-like configuration of the ceratobranchial 1, presence of the ceratobranchial 2, and presence of the epibranchial. First, presence of the paraglossal in dinosaur is ambiguous because, among extant reptiles, the paraglossal is present only in birds, while, absent in crocodylians and the other reptiles[17]. Moreover, medial groove of lateral margin of the "paraglossal" was not discussed previously in terms of function and homology with other hyoids in birds and absent in the paraglossal of neognath birds (Fig. 4 and Supplementary Fig. 2e). Second, as Hill et al.[17] mentioned, morphology of the ceratobranchial 1 is generally rod shape as in the other dinosaurs (e.g. Li et al.[18]). Presence of the ceratobranchial 2 in dinosaurs is unlikely because both extant archosaurs, crocodylians and birds, have no ceratobranchial 2 (Supplementary Fig. 4). In crocodylians, calcified portions of basihyal are fused in midline (Supplementary Fig. 4) as in turtles[44] and mistakenly identified as the ceratobranchial 2 in the past studies[16]. Finally, the epibranchial is also absent in crocodylians and palaeognath birds, indicating its lack in non-avian dinosaurs. Our identification of the laryngeal elements solves these problems in identification and match with preserved position of the *Pinacosaurus* fossil, which indicates in situ condition of the fossil. The previous study has hypothesized convergent presences of the paraglossal, ceratobranchial 2, and epibranchial between ankylosaurs and birds[17], while our

identification of cricoid, arytenoid, and ceratobranchial in ankylosaurs is reasonable because those three hyolaryngeal elements are common in reptiles, including birds (Fig. 4). Therefore, the identification of this study is more accountable and less assumptive than the past identification by Hill et al.[17].

2. Full description of hyolaryngeal apparatus of *Pinacosaurus* (IGM1000/3186)

Both cricoids (Fig. 1e-h) are complete but remain unfused along the midline, while those in the ankylosaurid *Saichania* are fused into a single element[20]. This probably has to do with the somatic immaturity of IGM100/3186[17]. The cricoids are the largest bones of the hyolaryngeal elements. They are at least 64.7 mm wide which is about three fourths the width of the mandible. The cricoid is pointed anteriorly and widely expanded posteriorly, with a concave posterior margin, forming an arrow-head shape in dorsal view. The cricoid body forms the floor of the element and is thin along the midline and thickens laterally. The anterior tip of the cricoid projects beyond the arytenoids, which may be an attachment site for constrictor muscle of glottis like ostrich[10]. The lateral edge of the cricoid body curls dorsally to form the cricoid wing. A dorsomedially projecting thin lamina extends anteroposteriorly from the anterior apex of the cricoid body and ends medial to the cricoid wing. At the intersection of the cricoid lamina and wing, a shallow groove (approximately 2 cm long) forms an articular facet for the arytenoid (Fig. 1c). The dorsal surface above the groove has a rugose surface, indicative of joint cartilage for the arytenoid. This rugose articular surface of the firm yet mobile crico-arytenoid joint might support active horizontal rotation of the arytenoid by contraction of the dilator muscles to open glottis. Only in birds does the arytenoid exhibit a distinct joint surface proximally, instead of a connective tissue bridge as in other reptiles (Supplementary Figs. 2a and 3d). A minute foramen lies near the posterior edge of the cricoid body on each side (Fig. 1a and fig. S1d), similar to two posterior notches in the cricoid of *Alligator mississippiensis*[7] and the angular foramen on the cricoid in several birds (Supplementary Figs. 2c and 3b) and chicken embryos[21]. The arytenoid lies dorsal to the cricoid (Fig. 1a,b,e-h). It is anteroposteriorly long and bears short dorsolateral and long dorsomedial wings, that form a J-shape in cross-section (Fig. 1). The dorsomedial wing is thin, and its anterior third projects dorsally to form the arytenoid process as in modern turtles[21] and birds[10]. The arytenoid process is an attachment site for m. dilator laryngis which opens the glottis. In birds it

originates from the caudolateral ends of cricoid[10,11], while, in crocodylians it originates from the posterolateral margin of basihyal and cricoid[7,9]. The dilator muscle may have inserted to the posterior part of the cricoid wing. The arytenoid process of *Pinacosaurus* is not as prominent as that of *Saichania*[20] (Supplementary Fig. 3). This is probably due to its relative somatic immaturity. In birds the arytenoid process ossifies late in ontogeny[10]. The dorsolateral wing is less developed than the dorsomedial wing. Furthermore, the lateral surface of the dorsolateral wing bears a ridge with a rugose surface that articulates with a corresponding rugose surface lying at the constriction of the above mentioned cricoid groove (Fig. 1d). This forms the ridge-and-groove articulation between the cricoid and arytenoid. This unique morphology of the articular facet is not found in other reptiles including birds.

The paired ceratobranchials have proximal ends that lie medially along the midpoint axis of the body, while their distal ends splay laterally (Fig. 1a). This symmetrical arrangement of the ceratobranchials along the midline and the dorsal position of the arytenoid to the cricoid indicate that the hyolaryngeal apparatus is preserved in almost life position, although they are slightly displaced posteriorly. The ceratobranchials were placed close to each other and the distance between the proximal ends are about 2 mm. This is much smaller than the width of the cricoid. Ceratobranchial 1 is long, slender, and slightly curves ventrally (Fig. 1b,e-h). It is 36 mm long, about the half length of mandible, and about 70 % of cricoid length. Its proximal end is circular in cross-section and has a flat surface. The lateral side of the proximal portion of the shaft is concave. The distal end is elliptical in cross-section and slightly expanded. Another pair of hourglass-shaped bones, preserved lateral to the ceratobranchial 1, is an atlas rib, identical to those found in the holotypic specimens of *Pinacosaurus*[45] and *Saichania*[20] (Supplementary Fig. 3). The ceratobranchials are discovered posterior to the larynx unlike birds and crocodilians. However, both the larynx and the ceratobranchials are articulated or associated to the basihyal, so the basihyal morphology defines their relative positions to each other. For example, a gecko and a turtle show the larynx cranial to the ceratobranchial 1 (Supplementary Fig. S5).

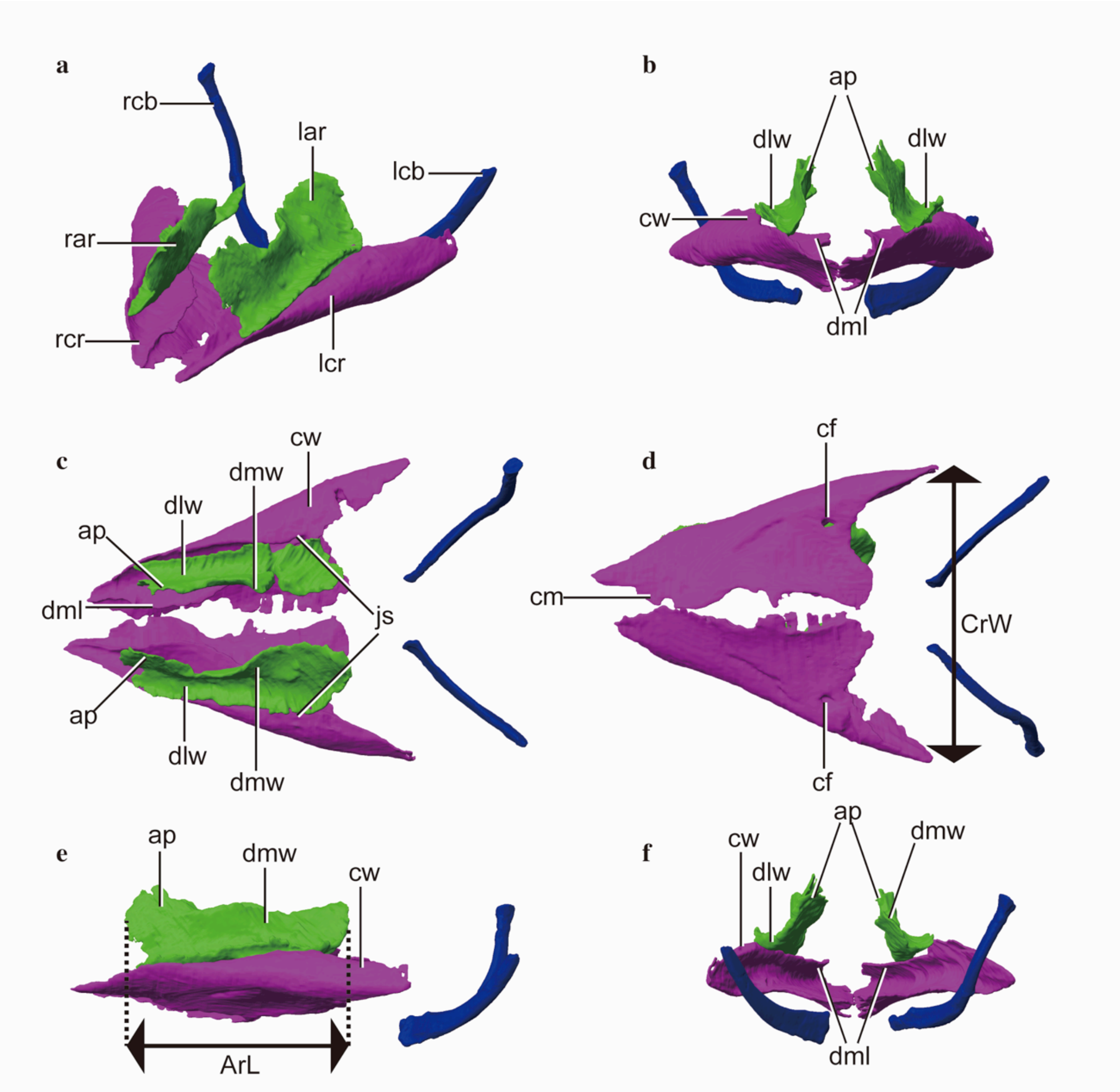

Supplementary Figure 1. 3D reconstructions of hyolaryngeal apparatus of *Pinacosaurus grangeri* (IGM100/3186). (**a**) Oblique, (**b**) anterior, (**c**) dorsal, (**d**) ventral, (**e**) left lateral, and (**f**) posterior views of the hyolaryngeal apparatus. Abbreviations: ar, arytenoid; ArL, arytenoid length; bh, basihyal; cb, ceratobranchial; cr, cricoid; CrW, cricoid width; eb, epibranchial: pcr, procricoid; ap, arytenoid process; cf, cricoid foramen; cm, coracoid midline; cw, cricoid wing; dlw, dorsolateral wing; dmw, dorsomedial wing; dml, dorsomedial lamina; and js, joint surface.

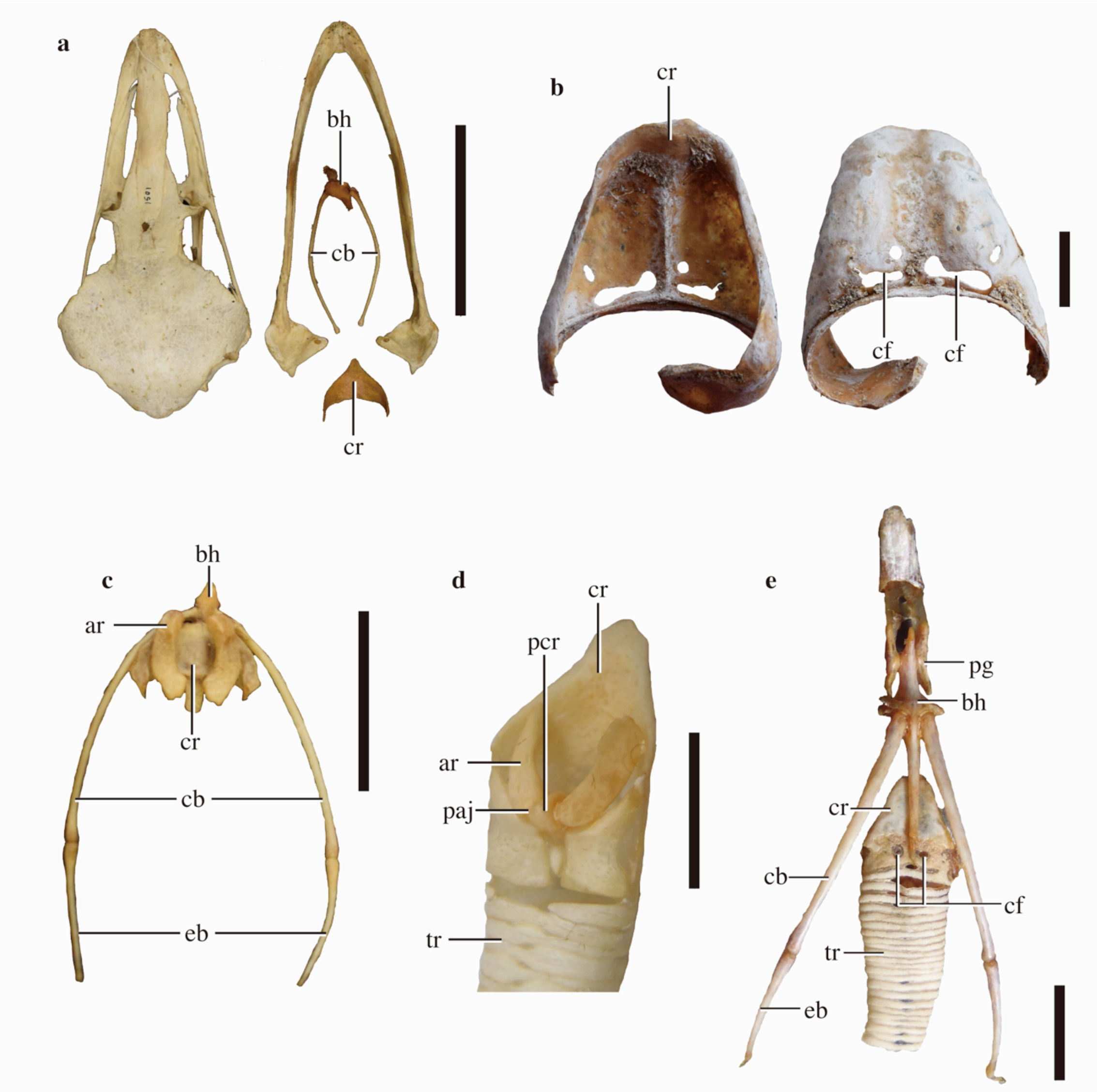

Supplementary Figure 2. Hyolaryngeal apparatus of birds. (**a**) *Struthio camelus* (AMNH1501). (**b**) *Dinornis* sp. (AMNH7301) in dorsal (left) and ventral views (right). (**c**) *Nothoprocta* sp. (AMNH6502). (**d**) *Phoenicopterus roseus* (NSMT AS100). (**e**) *Pandion haliaetus* (NSMT ASA1163). Scale bar is 10 cm in (**a**), 1cm in (**b**), (**c**), (**d**), and (**e**). Abbreviations: ar, arytenoid; bh, basihyal; cb, ceratobranchial; cr, cricoid; eb, epibranchial: pcr, procricoid; pg, paraglossal; paj, procricoid-arytenoid joint; and tr, tracheal ring.

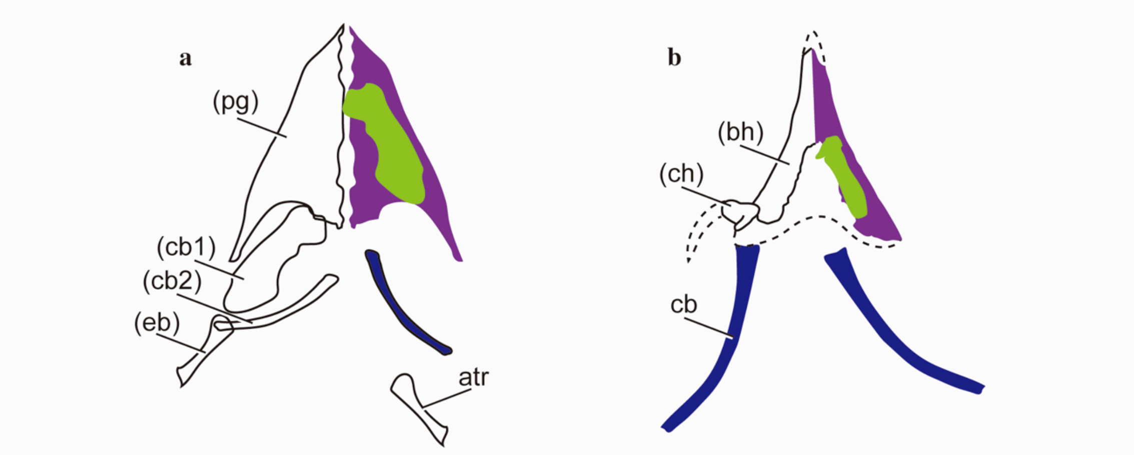

Supplementary Figure 3. Interpretations of hyolaryngeal apparatus of ankylosaurs. (**a**) *Pinacosaurus grangeri* (IGM100/3186). (**b**), *Saichania chulusanensis* (IGM 100/151). Cricoid is depicted in purple, arytenoid in green, and ceratobranchial in blue. Left half in (**a**) is based on Hill et al. (2015), and right half is an interpretation by this study. Likewise, left half in (**b**) is based on Maryanska (1977), and right half is an interpretation by this study. Abbreviations: atr, atlas rib; bh, basihyal; cb1, ceratobranchial 1; cb2, ceratobranchial 2; eb, epibranchial; and pg, paraglossal.

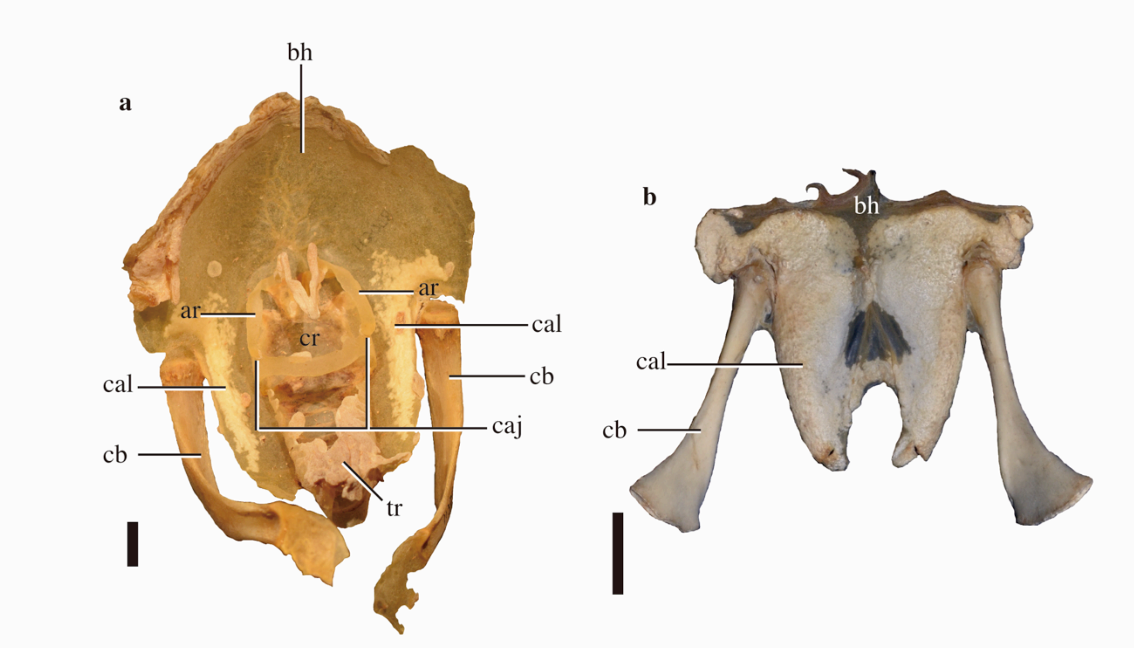

Supplementary Figure 4. Hyolaryngeal apparatus of modern crocodylians. (**a**) *Tomistoma schlegelii* (AMNH R113078). (**b**) *Caiman crocodilus* (ZSM70.1937). Scale bar is 1 cm. Abbreviations: ar, arytenoid; bh, basihyal; caj, cricoarytenoid joint; cb, ceratobranchial; cr, cricoid; eb, epibranchial: pcr, procricoid; pg, paraglossal; and tr, tracheal ring.

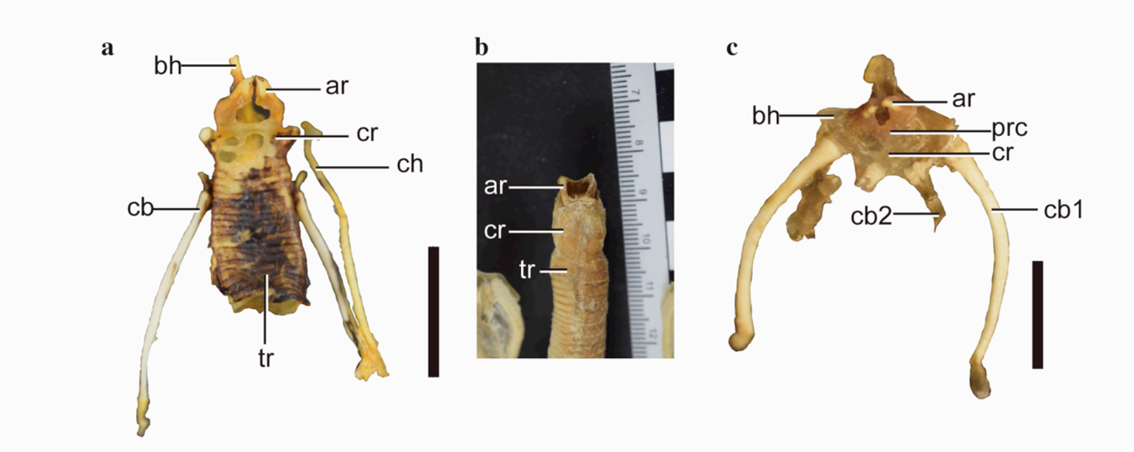

Supplementary Figure S5. Hyolaryngeal apparatus of non-archosaur reptiles (squamates and a turtle). (**a**) *Gekko gecko* (AMNH R154719). (**b**) *Varanus salvadorii* (AMNH R59873). (**c**) *Kinixys erosa* (AMNH R7210) showing partially-ossified arytenoids. Scale bar is 1 cm. Abbreviations: ar, arytenoid; bh, basihyal;caj, cricoarytenoid joint; cb, ceratobranchial; cr, cricoid; ch, ceratohyal; eb, epibranchial: pcr, procricoid; and tr, tracheal ring.

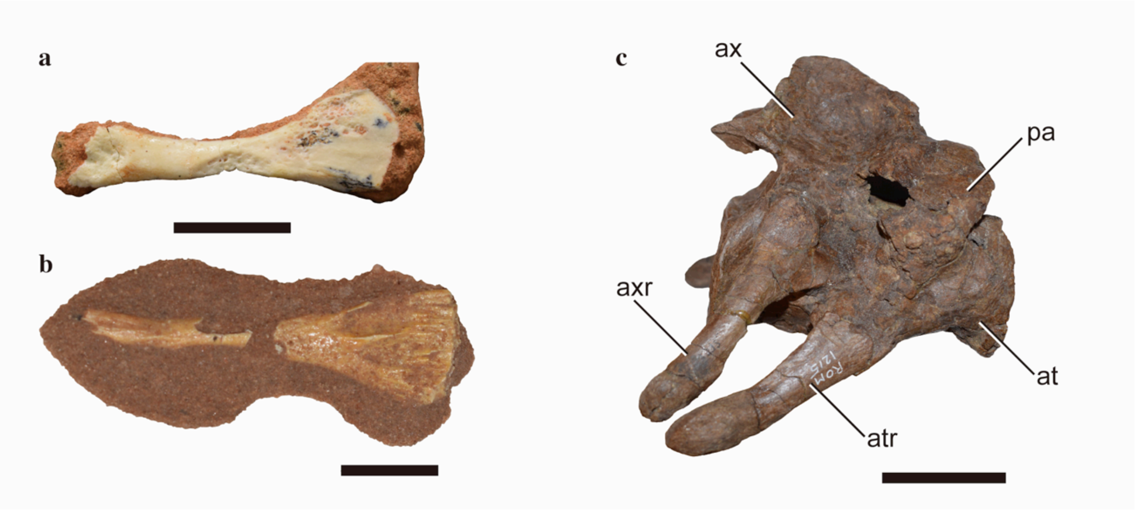

Supplementary Figure 6. Atlas ribs of ankylosaurs. (**a**) *Pinacosaurus grangeri* (IGM100/3186) in lateral view. (**b**) *Pinacosaurus grangeri* (AMNH 6523, holotype) in lateral view. (**c**) *Panoplosaurus* (ROM 1215) in lateral view. Abbreviations: at, atlas; ax, axis; atr, atlas rib; axr, axis rib; pa, proatlas. Scale bar is 1 cm in (**a**), (**b**), and 5 cm in (**c**).

References

44. Richter, S., Auer, M., Fritz, U. Variation of hyoid morphology in geoemydid terrapins. *Amphib.-reptil.* **28**, 148–153 (2007).

45. Gilmore, C. W. & Granger, W. Two new dinosaurian reptiles from Mongolia: with notes on some fragmentary specimens. *Am. Mus. Novit.* **679**, 1–22 (1933).

Supplementary Data (separate file). Morphometrics of larynx: arytenoid length, cricoid width, and the standardized values of those two measurements by mandible width.
