# Supplementary material for: An ankylosaur larynx provides insights for bird-like vocalization in non-avian dinosaurs
Source: Commun Biol. 2023 Feb 15;6:152. doi: 10.1038/s42003-023-04513-x (PMC9932143; doi:10.1038/s42003-023-04513-x)
Supplement: Supplementary file 2 — Description of Additional Supplementary Files [file 42003_2023_4513_MOESM2_ESM.pdf]

Description of Additional Supplementary Files

File name: Supplementary Data 1

Description: : The source data for Morphometrics of larynx.
