# Supplementary material for: An ankylosaur larynx provides insights for bird-like vocalization in non-avian dinosaurs
Source: Commun Biol. 2023 Feb 15;6:152. doi: 10.1038/s42003-023-04513-x (PMC9932143; doi:10.1038/s42003-023-04513-x)
Supplement: Supplementary file 4 — Reporting Summary [file 42003_2023_4513_MOESM4_ESM.pdf]

nature portfolio

Corresponding author(s): COMMSBIO-22-2483-T

Last updated by author(s): 2023/1/17

Reporting Summary

Nature Portfolio wishes to improve the reproducibility of the work that we publish. This form provides structure for consistency and transparency in reporting. For further information on Nature Portfolio policies, see our Editorial Policies and the Editorial Policy Checklist.

Statistics

For all statistical analyses, confirm that the following items are present in the figure legend, table legend, main text, or Methods section.

n/a	Confirmed	
☐	☒	The exact sample size (*n*) for each experimental group/condition, given as a discrete number and unit of measurement
☐	☒	A statement on whether measurements were taken from distinct samples or whether the same sample was measured repeatedly
☐	☒	The statistical test(s) used AND whether they are one- or two-sided<br>*Only common tests should be described solely by name; describe more complex techniques in the Methods section.*
☒	☐	A description of all covariates tested
☒	☐	A description of any assumptions or corrections, such as tests of normality and adjustment for multiple comparisons
☐	☒	A full description of the statistical parameters including central tendency (e.g. means) or other basic estimates (e.g. regression coefficient) AND variation (e.g. standard deviation) or associated estimates of uncertainty (e.g. confidence intervals)
☐	☒	For null hypothesis testing, the test statistic (e.g. *F*, *t*, *r*) with confidence intervals, effect sizes, degrees of freedom and *P* value noted<br>*Give P values as exact values whenever suitable.*
☒	☐	For Bayesian analysis, information on the choice of priors and Markov chain Monte Carlo settings
☒	☐	For hierarchical and complex designs, identification of the appropriate level for tests and full reporting of outcomes
☒	☐	Estimates of effect sizes (e.g. Cohen's *d*, Pearson's *r*), indicating how they were calculated

Our web collection on statistics for biologists contains articles on many of the points above.

Software and code

Policy information about availability of computer code

Data collection	Computed Tomography and Laser scanning are conducted in AMNH to study its morphology. We used CT images to make a reconstruction of the larynx from open data at Morphobank (http://www.morphobank.org, ID: P2101). extant species of reptiles and birds (45 specimens of turtles, 14 of lizards, 4 of crocodilians, and 90 of birds) are examined qualitatively and quantitatively in American Museum of Natural History, New York, and National Museum of Nature and Science, Tokyo (please see Supplementary Information). Maximum transverse widths of cricoid and mandible, and maximum anteroposterior lengths of arytenoid are measured (Supplementary Fig. 1). For size normalization, all the measurements were normalized using the regression equation (Supplementary Data). For relative size comparisons, residuals of arytenoid length and cricoid width are obtained from mandible width, which are all log-transformed variables (please see Supplementary Data).
Data analysis	All statistical analyses were performed using JMP Pro v.14.

For manuscripts utilizing custom algorithms or software that are central to the research but not yet described in published literature, software must be made available to editors and reviewers. We strongly encourage code deposition in a community repository (e.g. GitHub). See the Nature Portfolio guidelines for submitting code & software for further information.

Data

Policy information about availability of data

All manuscripts must include a data availability statement. This statement should provide the following information, where applicable:

- Accession codes, unique identifiers, or web links for publicly available datasets
- A description of any restrictions on data availability
- For clinical datasets or third party data, please ensure that the statement adheres to our policy

The datasets are available on the website of Communications Biology.

Human research participants

Policy information about studies involving human research participants and Sex and Gender in Research.

Reporting on sex and gender	*Use the terms sex (biological attribute) and gender (shaped by social and cultural circumstances) carefully in order to avoid confusing both terms. Indicate if findings apply to only one sex or gender; describe whether sex and gender were considered in study design whether sex and/or gender was determined based on self-reporting or assigned and methods used. Provide in the source data disaggregated sex and gender data where this information has been collected, and consent has been obtained for sharing of individual-level data; provide overall numbers in this Reporting Summary. Please state if this information has not been collected. Report sex- and gender-based analyses where performed, justify reasons for lack of sex- and gender-based analysis.*
Population characteristics	*Describe the covariate-relevant population characteristics of the human research participants (e.g. age, genotypic information, past and current diagnosis and treatment categories). If you filled out the behavioural & social sciences study design questions and have nothing to add here, write "See above."*
Recruitment	*Describe how participants were recruited. Outline any potential self-selection bias or other biases that may be present and how these are likely to impact results.*
Ethics oversight	*Identify the organization(s) that approved the study protocol.*

Note that full information on the approval of the study protocol must also be provided in the manuscript.

Field-specific reporting

Please select the one below that is the best fit for your research. If you are not sure, read the appropriate sections before making your selection.

☐ Life sciences ☐ Behavioural & social sciences ☒ Ecological, evolutionary & environmental sciences

For a reference copy of the document with all sections, see nature.com/documents/nr-reporting-summary-flat.pdf

Ecological, evolutionary & environmental sciences study design

All studies must disclose on these points even when the disclosure is negative.

Study description	The first report of the dinosaur larynx and its comparative analysis with modern reptiles and birds.
Research sample	Ankylosaur dinosaur Pinacosaurus (IGM100/3186) and extant species of reptiles and birds (45 specimens of turtles, 14 of lizards, 4 of crocodilians, and 90 of birds) in American Museum of Natural History, New York, and National Museum of Nature and Science, Tokyo.
Sampling strategy	This study presents the first attempt to measure the larynx of reptiles and birds, and a dinosaur.
Data collection	Junki Yoshida and Mark Norell collected the data in American Museum of Natural History, New York, and National Museum of Nature and Science, Tokyo.
Timing and spatial scale	The data were collected from 2018 to 2019.
Data exclusions	There is no excluded data.
Reproducibility	The measurement data used for this study are freely available as supplementary data of this manuscript.
Randomization	We did not use any statistics that would require randomization because the morphological data is taxonomically distinctive.
Blinding	Blinding was not applied for our study because the morphological data is taxonomically distinctive.

Did the study involve field work? ☐ Yes ☒ No

Reporting for specific materials, systems and methods

We require information from authors about some types of materials, experimental systems and methods used in many studies. Here, indicate whether each material, system or method listed is relevant to your study. If you are not sure if a list item applies to your research, read the appropriate section before selecting a response.

Materials & experimental systems

n/a	Involved in the study
☒	☐ Antibodies
☒	☐ Eukaryotic cell lines
☐	☒ Palaeontology and archaeology
☒	☐ Animals and other organisms
☒	☐ Clinical data
☒	☐ Dual use research of concern

Methods

n/a	Involved in the study
☒	☐ ChIP-seq
☒	☐ Flow cytometry
☒	☐ MRI-based neuroimaging

Palaeontology and Archaeology

Specimen provenance: The specimen access was permitted by the Mongolian Academy of Sciences.

Specimen deposition: The fossil specimen belongs to Geological Institute, Mongolian Academy of Sciences, Ulaanbataar, Mongolia

Dating methods: No new dating data are provided in this study.

☐ Tick this box to confirm that the raw and calibrated dates are available in the paper or in Supplementary Information.

Ethics oversight: No ethical approval is required for this study because we studied the specimens that are openly accessible.

Note that full information on the approval of the study protocol must also be provided in the manuscript.
